# Punishment-Induced Suppression of Methamphetamine Self-Administration Is Accompanied by the Activation of the CPEB4/GLD2 Polyadenylation Complex of the Translational Machinery

**DOI:** 10.3390/ijms26062734

**Published:** 2025-03-18

**Authors:** Atul P. Daiwile, Bruce Ladenheim, Subramaniam Jayanthi, Jean Lud Cadet

**Affiliations:** Molecular Neuropsychiatry Research Branch, DHHS/NIH/NIDA Intramural Research Program, 251 Bayview Boulevard, Baltimore, MD 21224, USA; atul.daiwile@nih.gov (A.P.D.); bladen@intra.nida.nih.gov (B.L.); jayanthi.sankar@va.gov (S.J.)

**Keywords:** methamphetamine, self-administration, DRD1 antagonist, CPEB, mRNA polyadenylation machinery

## Abstract

Methamphetamine (METH) use disorder (MUD) is a public health catastrophe. Herein, we used a METH self-administration model to assess behavioral responses to the dopamine receptor D1 (DRD1) antagonist, SCH23390. Differential gene expression was measured in the dorsal striatum after a 30-day withdrawal from METH. SCH23390 administration reduced METH taking in all animals. Shock Resistant (SR) rats showed greater incubation of METH seeking, which was correlated with increased *Creb1*, *Cbp*, and *JunD* mRNA expression. Cytoplasmic polyadenylation element binding protein 4 (*Cpeb4*) mRNA levels were increased in shock-sensitive (SS) rats. SS rats also showed increased protein levels for cleavage and polyadenylation specificity factor (CPSF) and germ line development 2 (GLD2) that are CPEB4-interacting proteins. Interestingly, GLD2-regulated GLUN2A mRNA and its protein showed increased expression in the shock-sensitive rats. Taken together, these observations identified CPEB4-regulated molecular mechanisms acting via NMDA GLUN2A receptors as potential targets for the treatment of METH use disorder.

## 1. Introduction

Methamphetamine (METH) use disorder (MUD) is a devastating, relapsing neuropsychiatric disease that is associated with enormous clinical, economic, and societal costs. Clinically, patients with MUD suffer from impaired emotional decision making [1,2] and deficits in impulsivity/reward processing and in social cognition [3]. Structural changes in brain areas involved in decision making and responsible for habitual actions have also been reported [4,5]. This is important because habitual behaviors are stimulus-driven, less dependent on present reward value, and are governed by behavioral automaticity observed in drug addiction [6,7]. Most importantly, overdose deaths of individuals who suffer from MUD in the United States have increased during the past decade [8,9].

As a first step towards elucidating the molecular basis of compulsive behaviors seen in substance use disorders (SUDs), our lab has modeled the DSM criterion of compulsive behaviors despite adverse consequences by using footshocks (punishment, adverse consequences), which are applied contingently during METH self-administration (SA) by rats [10,11,12,13,14,15,16]. In this model, punishment is able to separate rats into compulsive and non-compulsive METH takers [12]. Behavioral consequences resulting from compulsive drug SA are regulated, in part, by distinct but interacting brain areas that include the ventral tegmental area (VTA), substantia nigra (SN), nucleus accumbens, hippocampus, prefrontal cortex (PFC), amygdala, and the dorsal striatum [17,18,19,20]. We had therefore reasoned that compulsive METH taking might be regulated by dysfunctional molecular striatal pathways that drive this behavior [13,15,21,22] because the striatum receives large inputs from cortical and midbrain brain regions [23,24,25] that participate in reward circuitries [19]. To further explore the role of the dorsal striatum in compulsive METH taking and/or abstinence in the presence of adverse consequences, we have used a whole genome RNA sequencing approach to dissect the striatal transcriptional architecture related to punishment-associated compulsive or suppressed METH taking. This approach is important because only a small proportion of people who use amphetamine-type stimulants (ATS) develop stimulant use disorder [26].

The present study assessed the behavioral effects of the DRD1 antagonist SCH23390 on compulsive METH SA in the presence of footshocks. We also measured the propensity of the animals to relapse after 30 days of forced abstinence. In addition, we used RNA sequencing to identify potential transcriptional changes in the dorsal striatum after METH self-administration and withdrawal. Herein, we report that CPEB4 and its binding proteins might be involved in punishment-induced suppression of METH SA. CPEBs are translational regulators that are implicated in various biological functions [27,28] including the maintenance of local protein synthesis [29,30] as well as in hippocampus-dependent synaptic plasticity [31] and long-term memory [32]. There are four isoforms of the protein, CPEB1-4 [33], and they have been implicated in a variety of biological contexts, from translational regulation of embryonic cell division [34] to hippocampus-dependent behaviors [31]. Interestingly, CPEB1 and CPEB3 family members have been shown to participate in the maintenance of spatial memory that sustains cocaine-induced addiction-like behaviors [35]. Here, we report that non-compulsive individuals that had been exposed to METH showed significantly increased expression of the CPEB-binding protein, GLD2 (germline development defective 2), a post-transcriptional activator that mediates lengthening of the poly(A) tail [36] and participates in active translation [37]. GLD2 is also involved in regulating hippocampal synaptic plasticity [38,39]. The GLD2-regulated GLUN2A gene [39] also showed increased mRNA and protein levels. Together, these results implicate CPEB4/GLD2-dependent downstream signaling mechanisms in punishment-induced suppression of METH-taking behaviors and suggest the possibility of using them as targets for the treatment of MUD.

## 2. Results

### 2.1. Meth Self-Administration and Effects of Footshocks

**Figure 1:**
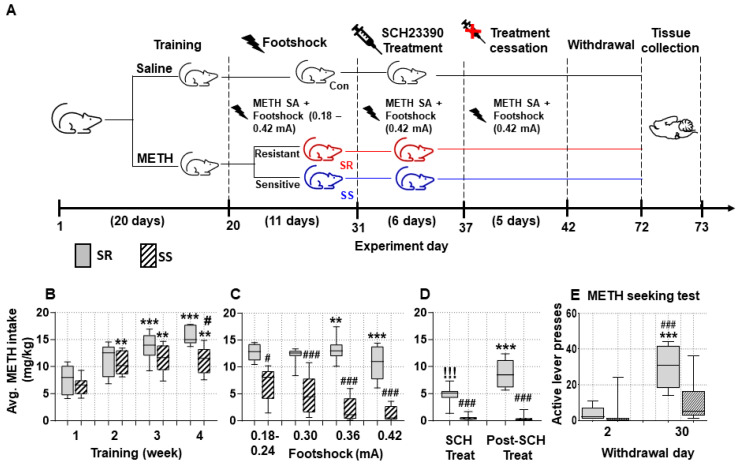
SCH23390 treatment results in decreased compulsive METH taking in a subpopulation of rats. (**A**) Experimental timeline showing METH SA sessions, the contingent administration of contingent footshocks, intraperitoneal SCH23390 treatments, and METH-seeking tests. Footshocks were administered randomly during 50% of pressing the active lever for METH. (**B**) During the first 4 weeks of METH SA, no significant differences were observed in average METH intake between METH groups, non-compulsive (SS) rats, *n* = 9, and compulsive (SR) rats, *n* = 7. (**C**) Footshocks caused a marked reduction in METH intake in the sensitive (SS) rats, whereas the resistant (SR) rats continued to self-administer METH. SR and SS rats were separated as per our previous published study in which we classified animals as shock-sensitive if they reduced their intake by 60% (10–16). (**D**) SCH23390 injections resulted in decreased average METH intake in SR and SS animals compared to their drug intake during the 0.42 mA footshock phase. Cessation of SCH23390 treatment resulted in higher average METH intake by SR rats. (**E**) SR rats showed greater incubation of METH craving on WD 30 in comparison to SS rats during the forced abstinence period. SR, shock-resistant; SS, shock-sensitive. Key to statistics, * *p* < 0.05, ** *p* < 0.01, *** *p* < 0.001, comparisons of average drug intake (1) First week of METH intake during the training phase with the second, third, and fourth weeks; (2) comparison of METH intake between 0.18 and 0.24 mA footshock with 0.30, 0.36, and 0.42; (3) comparison between SCH and Post-SCH treatment METH intake; and (4) active lever presses during WD2 and WD30, by SR and SS rats; # *p* < 0.05, ## *p* < 0.01, ### *p* < 0.001, comparisons between the SR and SS; ! *p* < 0.05, !! *p* < 0.01, !!! *p* < 0.001, comparison of METH intake between 0.42 mA footshock phase with the SCH treatment phase.

The time sequence of the behavioral experiment is shown in Figure 1A. As described previously [15], drug-naïve male Sprague Dawley rats (350–400 g) received either saline or METH (0.1 mg/kg/infusion) SA training under an FR1 schedule for 20 days. Then, contingent footshocks (0.18–0.42 mA) were introduced to the METH rats. METH self-administering and footshock-treated rats were separated post-facto into shock-resistant (SR)/compulsive (*n* = 7) and shock-sensitive (SS)/non-compulsive (*n* = 9) rats as described previously [10,11,12,13,14,15]. Specifically, animals were classified as shock-sensitive if they reduced their METH intake by 60% [10,11,12,13,14,15]. We observed significant effects of average (Avg) METH intake (F (3, 42) = 73.87, *p* < 0.0001) and Avg METH intake x Group interaction (F (3, 42) = 7.425, *p* = 0.0004), but a non-significant effect for Group (SR, SS) (F (1, 14) = 3.166, *p* = 0.0969) (Figure 1B), during the 4 weeks of METH SA training before the application of footshocks. Bonferroni’s post hoc test showed no significant differences in average METH intake between SR and SS rats during the first three weeks of training. However, METH intake of SS/non-compulsive rats was significantly lower than that of SR/compulsive animals (*p* = 0.0031) (SS: 11.24 mg/kg and SR: 15.53, respectively) during the fourth week of training (Figure 1B).

Following footshocks, there were significant effects of Group (F (1, 14) = 63.64, *p* < 0.0001), Avg METH intake (F (3, 42) = 15.05, *p* < 0.0001), and their interaction (F (3, 42) = 8.909, *p* = 0.0001) (Figure 1C). During the footshock phase, SR rats continue to take METH despite the adverse consequences, whereas SS rats decreased their METH intake (Figure 1C). Total METH intake for SR and SS rats on the first 2 days of shocks (0.18–0.24 mA) was 12.71 and 6.89 mg/kg, respectively (*p* < 0.05). However, with increasing intensity of footshocks (0.3, 0.36, and 0.42 mA), total quantities of METH taken for SS rats (5.09, 2.23, and 1.26 mg/kg, respectively) were lower than the SR rats (12.06, 13.25, and 10.93 mg/kg, respectively) (*p* < 0.001) (see Figure 1C).

### 2.2. The Effect of DRD1 Antagonist, SCH23390, on Compulsive METH-Taking Behaviors in the Presence of Punishment

There is, at present, no FDA-approved treatment for METH use disorder (MUD). Consistent with the observations by other investigators [40,41], we found that the DRD1 antagonist, SCH23390, can reduce METH intake in both compulsive and non-compulsive rats. Specifically, two-way ANOVA revealed significant effects for Group (F (1, 14) = 74.54, *p* < 0.0001), Avg METH intake (F (1, 14) = 54.97, *p* < 0.0001), and their interaction (F (1, 14) = 33.70, *p* = 0.0001) (Figure 1D). Compulsive rats consumed 11 mg/kg of METH prior to SCH23390 treatment but only 4.23 mg/kg of METH after SCH23390 treatment (*p* < 0.0001). Meanwhile, the METH intake of non-compulsive rats dropped from 1.26 to 0.52 mg/kg before and after SCH23390 treatment (*p* = 0.2440) (Figure 1D). We tested the duration of the effects of SCH23390 by stopping the drug while continuing footshocks. There were significant effects of Group (F (1, 14) = 93.05, *p* < 0.0001), Avg METH intake (F (1, 14) = 24.33, *p* = 0.0002), and their interaction (F (1, 14) = 26.74, *p* = 0.0001) (Figure 1D), with the compulsive rats increasing their METH intake from 4.23 to 8.74 mg/kg during that interval (*p* < 0.0001). In contrast, non-compulsive rats did not show any significant differences in their average METH intake (0.52 to 0.42 mg/kg) during and after stopping SCH23390 (see Figure 1D).

### 2.3. Prolonged Forced Abstinence of METH Showed Greater Incubation of METH Seeking in Compulsive Rats

Figure 1E illustrates the differences in cue-induced active lever presses in SR and SS rats after forced withdrawal of 1 month from METH SA. Two-way ANOVA revealed significant effects for Withdrawal Days (F (1, 14) = 29.48, *p* < 0.0001), Groups (F (1, 14) = 7.534, *p* = 0.0158), and their interaction (F (1, 14) = 8.720, *p* = 0.0105). Only SR rats showed significant increases in active lever presses on WD30 when compared to WD2 (Figure 1E).

Taken together, the behavior results suggested the existence of different molecular mechanisms that participate in regulating habit formation between the two METH SA phenotypes. In what follows, we describe our analysis of differential striatal gene expression in the compulsive and non-compulsive rats.

### 2.4. Global Transcriptional Profiling of Long-Term METH SA Reveals Differentially Expressed Genes (DEGs) in Compulsive and Non-Compulsive Rats

**Figure 2:**
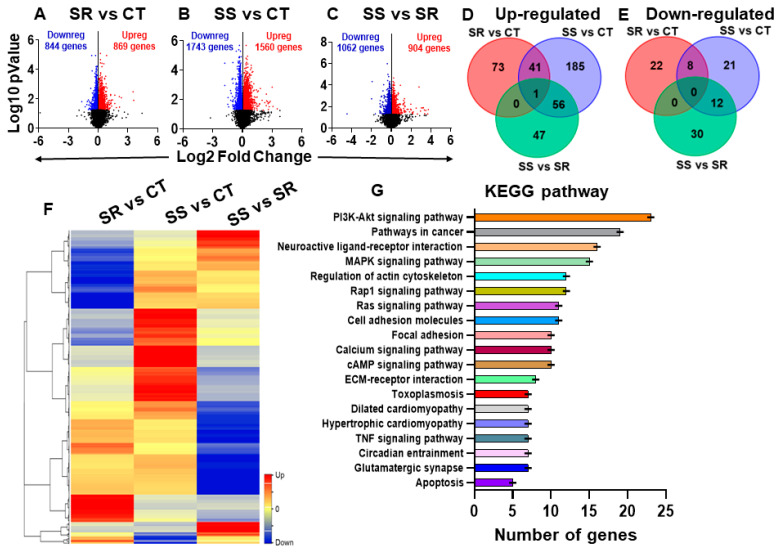
RNA sequencing revealed large-scale changes in gene expression in the dorsal striatum. Analysis of raw sequencing data using log2 fold changes and log10 *p*-values revealed many differentially expressed genes in pairwise comparisons shown as volcano plots: (**A**) SR vs. CT, (**B**) SS vs. CT, and (**C**) SS vs. SR. The Venn comparison was shown in (**D**) up-regulated and (**E**) down-regulated genes. (**F**) Hierarchical clustering of differentially expressed genes across 3 pairwise comparisons. The red color represents over-expressed genes, the blue color represents genes with reduced expression, while the yellow color represents genes with no changes in expression. (**G**) KEGG analysis shows the pathway distribution of differentially expressed genes according to DAVID.

**Figure 3:**
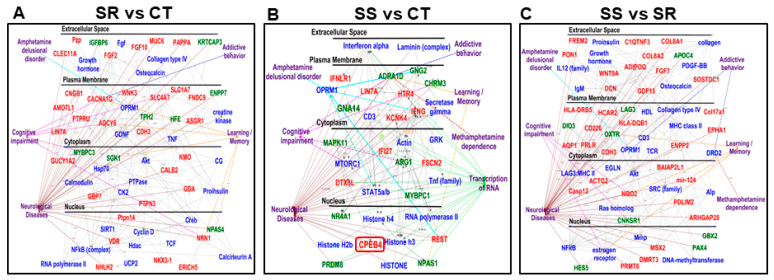
Ingenuity pathway analysis (IPA) (**A**) SR vs. CT, (**B**) SS vs. CT, and (**C**) SS vs. SR identifies a molecular network that differentially expressed genes are involved in addiction, drug dependence, cognitive impairment, learning, and memory (the red color indicates upregulated genes, the green color represents downregulated genes, and the blue color represents interacting gene partners).

We used RNA sequencing to identify global transcriptional changes in the dorsal striatum, which may be associated with METH SA and the incubation of drug craving. The results of the RNA sequencing are shown in Figure 2. Using DESeq2 (version 1.47.5), we performed three pairwise comparisons (SR vs. CT, SS vs. CT, and SS vs. SR) (Figure 2A–C). Analysis of raw RNA sequencing data using log2 fold changes and log10 *p*-values, illustrated as volcano plots, identified 1713 statistically significant differentially expressed genes (DEGs) in the SR rats (Figure 2A) and 3303 DEGs in the SS rats (Figure 2B) in comparison to control rats. The comparison between the SS and SR groups revealed a total of 1966 DEGs (Figure 2C). We then used a more restrictive cut-off of equal to or greater than 1.45-fold (*p* = 0.05) for the three pairwise comparisons in order to make better sense of the data. The decreased number of DEGs that resulted from these constraints is provided in the Venn diagrams (Figure 2D,E). We also provided a hierarchical cluster analysis (Figure 2F) to show the relationship of 487 genes shown in the Venn diagrams in Figure 2D,E. The cluster reveals that different sets of genes are impacted in the compulsive and non-compulsive rats, with genes that are highly up-regulated in the compulsive rats being down-regulated in the non-compulsive rats and vice versa (Figure 2F). The Database for Annotation, Visualization, and Integrated Discovery (DAVID) (version v2023q4) was used to gain insight into the possible functional biological pathways involved with the DEGs shown in the cluster. They included genes that participated in glutamatergic signaling, neurobiologically active ligand-receptor interactions, regulation of the actin cytoskeleton, the cyclic AMP signaling pathway, and the calcium signaling pathway, among others (Figure 2G). The Qiagen ingenuity pathway analysis (IPA) software (v01-23-01) allowed us to identify gene networks that may have contributed to the behaviors associated with METH SA and withdrawal. IPA identified several DEGs with involvement in the manifestations of cognitive impairments, learning/memory, addictive behavior, METH dependence, and delusional disorder (Figure 3A–C).

### 2.5. Specific Striatal Genes Are Differentially Expressed in Rats Prone to Relapse

**Figure 4:**
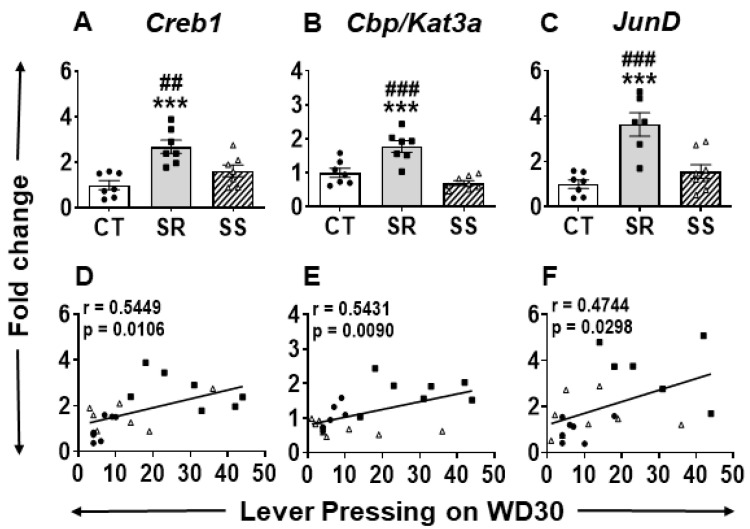
Increase mRNA expression of (**A**) *Creb*, (**B**) *Kat3a/Cbp*, and (**C**) *JunD* in the dorsal striatum of SR rats compared to CT and SS. Regression analysis revealed a positive correlation between active lever responding on WD30 and fold change for (**D**) *Creb*, (**E**) *Kat3a/Cbp*, and (**F**) *JunD*. CT, saline; SR, shock-resistant; and SS, sensitive rats. Key to statistics, * *p* < 0.05, ** *p* < 0.01, *** *p* < 0.001, comparisons between METH groups (SR and SS) and controls (CT); # *p* < 0.05, ## *p* < 0.01, ### *p* < 0.001, comparison between SR and SS.

One of the obstacles that works against the development of effective treatment against METH use disorder (MUD) is frequent relapse episodes. In the current study, only the compulsive rats showed incubation of METH craving after forced withdrawal from METH SA and punishment (Figure 1E). We reasoned that DEGs that participate in this phenomenon would be found only in the compulsive rats in comparison to the other two groups. These consisted of 95 DEGs in the SR vs. CT comparison and 77 DEGs in the SS vs. SR comparison. Of these DEGs, quantitative PCR showed that compulsive rats showed significantly higher mRNA levels for cAMP-response element binding protein (Creb1) (F (2, 18) = 11.27, *p* = 0.0007) (Figure 4A), CREB binding protein (Cbp/Kat3a) (F (2, 19) = 19.37, *p* < 0.0001) (Figure 4B), and JunD (F (2, 18) = 15.23, *p* = 0.0001) (Figure 4C) in comparison to control and non-compulsive rats. Importantly, regression analysis confirmed comparable positive correlations for *Creb1* (r = 0.5449, *p* = 0.0106), *Cbp/Kat3a* (r = 0.5431, *p* = 0.0090), and *JunD* (r = 4744, *p* = 0.0298) (Figure 4D–F, respectively) between active lever presses on WD30 and mRNA levels.

### 2.6. Non-Compulsive METH Takers Show Differential Expression of CPEB4

Frequent relapses of compulsive METH seeking and taking during intervals of abstinence are an important obstacle to MUD treatment. These behavioral patterns are probably related to molecular adaptations in the brains of patients who relapse. Other METH users who do not relapse might have different transcriptional neuroadaptations. Here, we indeed found different global transcriptional changes in the dorsal striatum of non-compulsive rats in comparison to control and compulsive rats in SS rats. These might be due to alterations in the expression of transcription factors and/or mRNA stability machinery [42]. As seen in Figure 3B, this reasoning is consistent with the involvement of the cytoplasmic polyadenylation element-binding protein 4 (Cpeb4) [28,43] in the gene networks that are related to cognitive impairment, learning/memory, METH dependence, and delusional and neurological disorder. Indeed, the mRNA poly(A) tail, which plays a critical role in the control of mRNA stability and translation [44], is regulated by proteins that belong to the cytoplasmic polyadenylation element-binding protein (CPEB) family [28,43,45,46,47].

**Figure 5:**
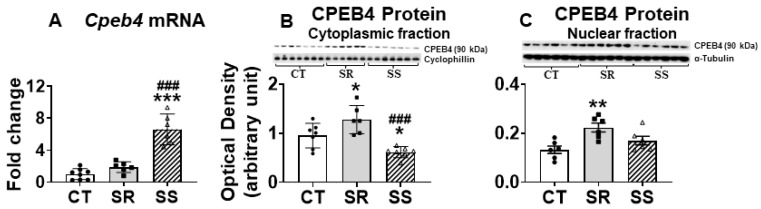
*Cpeb4* mRNA and protein levels in the dorsal striatum of compulsive and non-compulsive rats. (**A**) PCR validation of RNA sequencing data identified increased expression in *Cpeb4*. (**B**) Cytoplasmic protein analysis by Western blot showed increased levels of CPEB4 in SR but decreased levels in SS rats. (**C**) Nuclear protein analysis showed increased levels of CPEB4 in SR and SS rats. Keys to statistics are as described in Figure 4. * *p* < 0.05, ** *p* < 0.01, *** *p* < 0.001, ### *p* < 0.001.

We used q-PCR to validate the findings of up-regulated *Cpeb4* (F (2,19) = 5.332, *p* = 0.0145) in the non-compulsive rats (Figure 5A). Unexpectedly, Western blot analysis revealed lower cytoplasmic CPEB4 protein expression (F (2,18) = 13.34, *p* = 0.0003) in the sensitive rats in comparison to CT and resistant rats (Figure 5B). Because regulation of RNA processing and translational control depends on shuttling of CPEBs between the nucleus and cytoplasm [48,49], we also measured CPEB4 in the nuclear fraction and found non-significant increases (30%) in its protein levels in the sensitive rats (Figure 5C). We decided to also measure the mRNAs for other members of the CPEB family (*Cpeb1*, *Cpeb2*, and *Cpeb3*). There were no changes in *Cpeb1* mRNA, but significant decreases in CPEB1 protein levels [F (2,18) = 45.46, *p* < 0.0001] in both compulsive and non-compulsive rats (Figure S1A,B), suggesting that exposure to METH was enough to impact striatal CPEB1 protein expression. In addition, the sensitive (SS) rats exhibited increased *Cpeb2* mRNA levels (F (2,17) = 8.168, *p* = 0.0033) in comparison to the other two groups, with there being decreased protein levels (F (2,19) = 4.443, *p* = 0.0261) only in the resistant (SR) animals compared to controls (Figure S1C,D). SR and SS rats displayed no significant changes in *Cpeb3* mRNA expression, whereas SR rats showed significantly increased CPEB3 protein levels (F (2,19) = 9.595, *p* = 0.0013) (Figure S1E,F).

### 2.7. Altered CPEB4 Expression in Non-Compulsive METH-Taking Rats Is Associated with Changes in the Expression of CPEB4-Interacting Proteins

**Figure 6:**
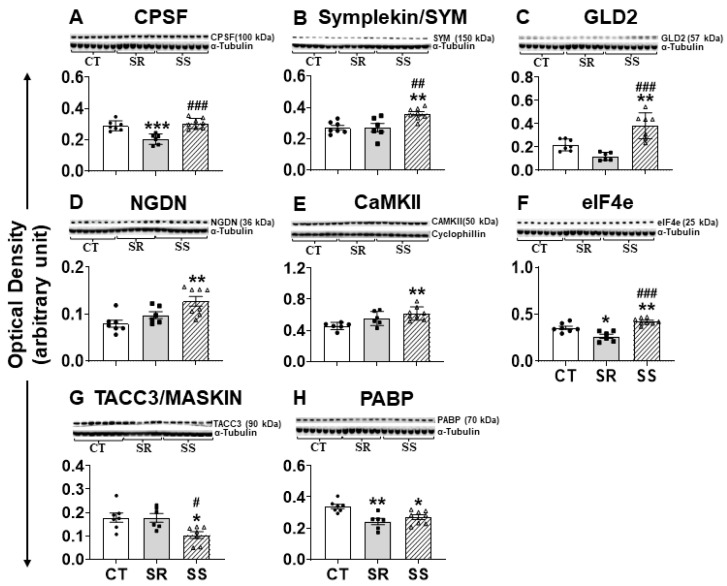
Protein expression analysis of members of the mRNA polyadenylation complex. (**A**) CPSF, (**B**) Symplekin, (**C**) GLD2, (**D**) NGDN, (**E**) CaMKII, (**F**) elF4e, (**G**) TACC3/Maskin, and (**H**) PABP. Keys to statistics are as described in Figure 4. * *p* < 0.05, ** *p* < 0.01, *** *p* < 0.001, # *p* < 0.05, ## *p* < 0.01, ### *p* < 0.001.

CPEBs exert their effects by interacting with several proteins [28,43]. Figure 6 shows that the protein expression of several CPEB4-interacting proteins was impacted after METH exposure. These included cleavage and polyadenylation specificity factor (CPSF), which binds to the cis element in the RNA essential for polyadenylation; symplekin (SYM), a scaffold protein that helps link CPEB to CPSF; germ line development 2 (GLD2), a cytoplasmic poly(A) polymerase; neuroguidin (NGDN); and poly(A) ribonuclease (PARN) [27,50]. CPSF showed significant decreases (F (2,18) = 18.07, *p* < 0.0001) in the SR rats in comparison with the CT and SS groups (Figure 6A). In addition, SYM (F (2,18) = 8.812, *p* = 0.0021) and GLD2 (F (2,17) = 20.54, *p* < 0.0001) showed significant increases in the SS rats in comparison to CT and SR rats (Figure 6B,C). Moreover, NGDN protein levels (F (2,18) = 7.966, *p* = 0.0033) showed significant increases in SS rats only in comparison to CT rats but not to SR rats (*p* = 0.0781) (Figure 6D). There were no significant changes in protein levels of PARN in any group of rats (Figure S2A).

The mRNAs with short poly(A) tails continued to be in a translationally inactive state by the protein–protein interaction complex of CPEB-maskin-eIF4E. Upon stimulation by CaMKII/AURKA/CDK1, CPEBs recruit protein complexes, and short poly(A) tail mRNA undergoes cytoplasmic polyadenylation with the help of GLD2, which leads to increased binding of PABP to eIF4G, which helps dislocate maskin from eIF4E, thereby allowing the commencement of translation [51]. There was a significant increase in the protein expression for CaMKII (F (2,17) = 7.372, *p* = 0.005) in the SS rats in comparison to CT rats (Figure 6E), but the levels of AURKA and CDK1 remain unaffected (Figure S2B,C). Other CPEB4-related proteins of interest included elF4E, which showed significant increases (F (2,18) = 21.22, *p* < 0.0001) (Figure 6F), and maskin/TACC3, whose protein levels were significantly decreased (F (2,17) = 5.985, *p* = 0.0108) (Figure 6G) in the SS rats compared to SR and control rats. Furthermore, PABP protein levels were also significantly decreased (F (2,18) = 9.108, *p* = 0.0019) in SR and SS rats compared to CT (Figure 6H).

**Figure 7:**
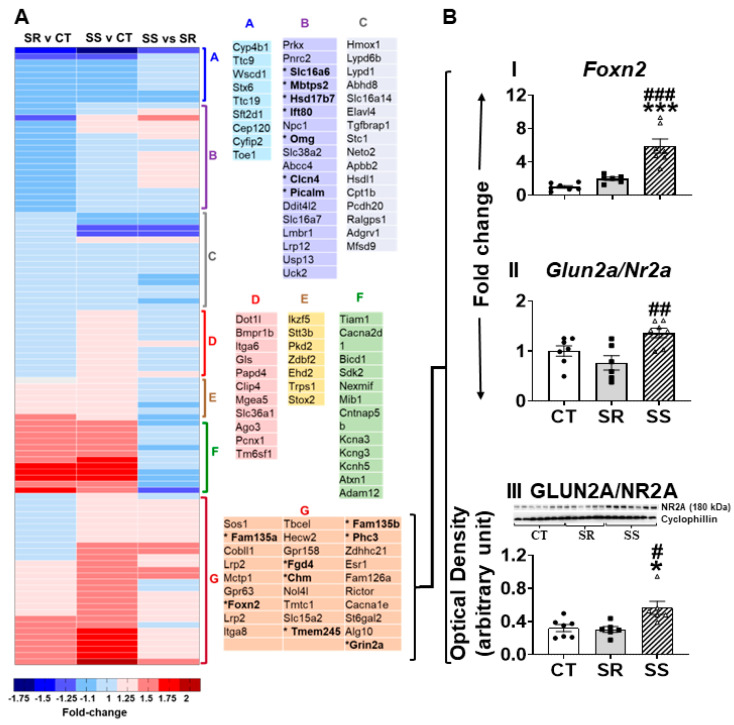
Identification of GLD2-regulated genes according to Udagawa et al. (2012) [39] as revealed in our RNA sequencing data. (**A**) Hierarchical clustering of GLD2-regulated genes in RNA sequencing data across 3 pairwise comparisons. The red color represents over-expressed genes, the blue color represents genes with reduced expression, while the yellow color represents genes with no changes in expression. (**B**) PCR validation of RNA sequencing data identified increased expression of (I) *Foxn2* and (II) *Glun2a*, (III) GLUN2A protein levels by Western blot analysis. Keys to statistics are as described in Figure 4. * *p* < 0.05, *** *p* < 0.001, # *p* < 0.05, ## *p* < 0.01, ### *p* < 0.001.

As shown above, GLD2 protein levels were increased in the SS rats (Figure 6C). GLD2 belongs to a family of poly(A) polymerases that protect mRNAs from deadenylation, thereby increasing their stability [52,53,54,55]. GLD2 is also a known regulator of biological processes involved in development and gametogenesis [27,52,56]. Moreover, GLD2 participated in regulating synaptic plasticity and long-term memory formation [52,53,57] by regulating gene expression [39]. We thus seek to identify potential GLD2 targets [39] in our RNA sequencing analysis in order to determine if GLD2 targets genes might also participate in promoting shock-induced suppression in our model (Figure 7A). Using quantitative RT-PCR, we were able to confirm upregulation for *Foxn2* (F (2,17) = 24.32, *p* < 0.0001) and *Glun2a* (F (2,17) = 6.849, *p* = 0.0066) in the SS rats (Figure 7B(I,II)). Because the glutamatergic system is important in neuroadaptive changes caused by stimulants [58,59], and GLUN2A surface protein expression during synaptic plasticity is mediated through CPEB and CPEB-associated factors GLD2 and NGDN [39,57], we decided to measure its protein expression. We also found that GLUN2A protein levels were significantly increased (F (2,16) = 6.869, *p* = 0.0070) in the abstinent rats in comparison to the control and compulsive (SR) groups (Figure 7B(III)).

## 3. Discussion

In the present study, we sought to assess the behavioral effects of the DRD1 antagonist SCH23390 on compulsive METH SA in the presence of footshocks. We also measured the propensity of relapse after 30 days of forced abstinence. Moreover, we attempted to identify potential changes in striatal gene expression after METH self-administration and withdrawal using RNA sequencing. Administration of the DRD1 antagonist, SCH23390, to METH self-administering rats reduced drug intake in both compulsive (SR) and non-compulsive (SS) phenotypes. Interestingly, compulsive, but not SS, rats increased their METH intake when administration of the DRD1 antagonist (SCH23390) was stopped. Incubation of METH craving was prominent in compulsive but not in non-compulsive individuals. RNA sequencing data identified genes like *Creb1*, *Cbp*, and *JunD* whose mRNA levels were impacted differentially in the compulsive rats. Differential changes in gene expression were also observed in the non-compulsive rats. These included CPEB4, which showed increased mRNA levels in these rats. Below, we discuss the potential relevance of these changes in footshock-induced suppression of METH SA.

### 3.1. Effects of the DRD1 Antagonist on Compulsive METH SA

There is no FDA-approved medication available to treat METH use disorder (MUD). In the present study, we found that SCH23390, a DRD1 antagonist, effectively suppressed METH taking in both non-compulsive and compulsive rats. Our current finding agrees with that of a previously published study wherein [40] had found that intra-striatal administration of SCH23390 reduced METH SA in rats. Together, these results suggest the need to better evaluate drugs that inhibit DRD1 as potential therapeutic agents against MUD.

### 3.2. Compulsive METH-Taking Rats Are Prone to Relapse

Compulsive SR rats that showed higher METH intake during the punishment (footshock) phase also displayed higher incubation of drug craving than shock-sensitive rats. Those results are consistent with our previous publications [10,11,12,13]. We measured potential transcriptional changes in the dorsal striatum because of its participation in different aspects of compulsive drug-taking behaviors and withdrawal from METH [15,16,60,61,62]. Higher propensity to relapse shown by SR animals is related to changes in the expression of *Creb1*, *Cbp*, and *JunD* (Figure 4A–F). The changes in Creb1 and Cbp mRNA levels are consistent with our previous data identifying CREB-mediated dependent mechanisms as mediators of METH-induced transcriptional changes in the striatum [60,63,64,65,66].

### 3.3. CPEB and Dynamic Transcriptional Reprogramming in Non-Compulsive Animals

RNA sequencing identified increased expression of Cpeb4 in the non-compulsive rats. CPEB4 is an RNA-binding protein that regulates the translation of approximately 40% of mRNA transcripts that hold cytoplasmic polyadenylation element (CPE) sequences in their 3′ UTRs [67]. This impact of CPEB4 might explain, in part, the results of the IPA that revealed the potential involvement of CPEB4 in transcription, cognitive impairment, learning/memory, addictive behavior, METH dependence, and delusional and neurological disorder. It is noteworthy that increased *Cpeb4* mRNA [68] and CPEB4 protein levels [35] were reported in the prefrontal cortex and striatum of animals exposed to cocaine, thus supporting its potential role in the behavioral consequences of psychostimulant exposure.

Regulation of RNA processing and translational control depends on shuttling of CPEBs between the nucleus and cytoplasm [49]. Both overstimulation of N-methyl D-aspartate (NMDA) receptors and endoplasmic reticulum calcium depletion induce CPEB4 nuclear retention [48]. Among the critical roles played by NMDA receptors in synaptic plasticity, there has been some focus on the translational regulation of the GLUN2A subunit because of its dendritic localization [39] and its activation during long-term potentiation [69,70]. It is therefore noteworthy that we detected increased GLUN2A protein levels in the non-compulsive rats, results that appear to be related to CPEB/GLD2-dependent mechanisms.

## 4. Materials and Methods

### 4.1. Animals

Male Sprague Dawley rats weighing 350–400 g were purchased from Charles River Labs (Kingston, New York, NY, USA) and habituated, in a 12 h reverse-light dark cycle, for 7–15 days prior to surgery. Intravenous surgery was performed as per our previous publications [10,11,15]. More details are provided in Supplementary Text Section S1.

### 4.2. Drugs

Methamphetamine HCl (+, −) (NIDA Drug Supply Program, Baltimore, MD, USA) was dissolved in 0.9% NaCl at a concentration of 0.1 mg/mL. The dopamine receptor D1 (DRD1) antagonist, SCH-23390 (+, −) hydrochloride (Research Biochemicals, Natick, MA, USA), was mixed in saline and injected intraperitoneally in a volume of 1 mL/kg, 30 min prior to each METH SA session.

### 4.3. METH Self-Administration and Foothocks

Drug-naive rats were allowed to self-administer METH (0.1 mg/kg/infusion) and saline during three 3 h sessions (9 h/day) with a 30 min time interval between each session using an FR1 schedule [10,11,12,13,14,15]. There was a 20 s timeout between each infusion as described in our previous papers. After rats had escalated their intake of METH, they received increased intensity of contingent footshocks that led to their segregation into shock-resistant and shock-sensitive rats [12]. The detailed METH SA training procedures are described in the Supplementary Text, Section S2. The time sequence for the various phases of behavioral experiments is shown in Figure 1A.

### 4.4. DRD1 Antagonist, SCH23390, Treatment During METH SA, and Footshock Punishment

Immediately after footshocks, METH rats were separated into SR and SS phenotypes. Only METH self-administering rats received intraperitoneal injections of different doses of SCH23390 (0, 0.1, 0.25, and 0.5 mg/kg), 30 min before each behavioral session following a Latin-square design for 6 days as described in the previous publication [15]. Contingent footshocks were continuously applied during the SCH23390 treatment period. Saline self-administering rats received saline injections (i.p.). Saline rats were never subjected to footshock.

Resurgence phase after stopping the administration of the DRD1 antagonist. After we stopped the SCH23390 treatment, we continued the behavioral experiment in the presence of contingent footshocks (0.42 mA). Thereafter, we measured METH SA for 5 days.

### 4.5. Measurements of Incubation of METH Craving

At the end of the various phases of the behavioral experiment (Figure 1), rats were removed from the experimental boxes and transferred to their home cages located in the animal vivarium for 30 days. During that time, rats were tested for METH seeking on withdrawal day 2 (WD2) and WD30. More details are provided in the Supplementary Text, Section S3.

### 4.6. RNA Extraction and Sequencing

Rats were euthanized 24 h after the drug-seeking test on WD30 using rapid decapitation with a guillotine. Rat dorsal striata were dissected out using specific neuroanatomical coordinates (A/P + 2 to −2 mm bregma, M/L ± 2 to 5 mm, D/V −3 to −6 mm) and immediately snap-frozen on dry ice and stored at −80 °C. Total RNA was extracted from the dorsal striatum using the Qiagen RNeasy Mini kit (Qiagen, Valencia, CA, USA), and RNA integrity (RIN) was checked using the Agilent bioanalyzer 2100, and six samples per group with RIN 8 or above were shipped on dry ice to Azenta, Genewiz (Waltham, MA, USA) for RNA sequencing. More details for the analysis of the RNA sequencing data are provided in the Supplementary Text, Section S4, and in the results section below. The RNA-Seq data have been deposited in NCBI under GEO accession number GSE200564.

### 4.7. Quantification of mRNA Levels by qRT-PCR Analysis

Total RNA (500 ng) was reverse-transcribed using the Advantage RT-for-PCR kit (Clontech, Mountain View, CA, USA). Quantitative RT-PCR analyses were carried out using Roche LightCycler 480 II with Luna Universal qPCR SYBR GREEN (NEB Inc., Ipswich, MA, USA) according to the manufacturer’s protocol. We purchased gene-specific primers from the Synthesis and Sequencing Facility of Johns Hopkins University (Baltimore, MD, USA). These were generated using Thermo Fisher Scientific (Waltham, MA, USA) (OligoPrefect Primer Designer software) (version v.7). Relative amounts of mRNA were normalized using beta-2 microglobulin (B2m) mRNA as the standard. The primer sequences used for RT-PCR are listed in Table S1.

### 4.8. Western Blot

Dorsal striatal tissues from the other hemisphere were homogenized using 10 mM Tris HCl, 150 mM NaCl, pH 7.5 in the presence of 1% Nonidet P-40 (NP-40) protein and phosphatase inhibitor cocktails (Sigma, St. Louis, MO, USA). Total protein concentrations were quantified using the BCA assay (Thermo Fisher Scientific, Waltham, MA, USA). Protein levels of CPEB1, CPEB2, CPEB3, CPEB4, CAMKII-α, eIF4e, GLD2, AUORA-A, TACC3, PABP, CDK1, CPSF2, PARN, symplekin, and NR2A were analyzed in the rat dorsal striatum. Cyclophillin and α-tubulin were used as reference controls. More details are provided in the Supplementary Text (Section S5).

### 4.9. Statistical Analysis

Behavioral, mRNA, and protein data were analyzed with the statistical program GraphPad Prism (version 9, GraphPad software, La Jolla, CA, USA). Detailed statistical analyses are described in the Supplementary Text (Section S6).

## 5. Conclusions

Our study reports, for the first time, the involvement of the mRNA polyadenylation machinery in the suppression of METH craving-like behaviors after long-term forced abstinence. We also found that rats that showed incubation of METH craving showed increased *Creb1*, *Cbp*, and *JunD* mRNA expression that correlated with the number of active levers pressing after prolonged abstinence. In contrast, there were increases in the *Cpeb4* mRNA levels in the dorsal striatum of rats whose METH-taking behaviors were suppressed by footshocks. The increased GLD2 expression in the non-compulsive rats suggests that CPEB4/GLD2-dependent mechanisms might be important determinants of suppression of METH intake, apparently, via increased expression of the glutamate receptor subunit, GLUN2A/NR2A, whose expression is also altered during synaptic plastic changes in the brain. Targeting the mRNA polyadenylation complex may constitute a novel avenue for therapeutic interventions against METH use disorder.

## Data Availability

The RNA sequencing data have been deposited at the NCBI GEO under the accession # GSE200564. All other data generated in this study will be made available upon reasonable request to the corresponding author via email.

## Supplementary Materials

The following supporting information can be downloaded at https://www.mdpi.com/article/10.3390/ijms26062734/s1.
