# Supplementary material for: Punishment-Induced Suppression of Methamphetamine Self-Administration Is Accompanied by the Activation of the CPEB4/GLD2 Polyadenylation Complex of the Translational Machinery
Source: Int J Mol Sci. 2025 Mar 18;26(6):2734. doi: 10.3390/ijms26062734 (PMC11942873; doi:10.3390/ijms26062734)
Supplement: Supplementary file 1 [file ijms-26-02734-s001.zip › ijms-3456888-supplementary.pdf]

International Journal of *Molecular Sciences*

MDPI

Supplementary Materials for

Punishment-Induced Suppression of Methamphetamine Self-Administration Is Accompanied by the Activation of the CPEB4/GLD2 Polyadenylation Complex of the Translational Machinery

Atul P. Daiwile [†], Bruce Ladenheim [†], Subramaniam Jayanthi and Jean Lud Cadet *

[†] These authors contributed equally.
* Correspondence: jcadet@intra.nida.nih.gov

Supplementary Text (Materials and Methods)

1. Animals

Male Sprague Dawley rats weighing 350–400 g were purchased from Charles River Labs (Raleigh, NC, USA) and group-housed in light (12 h reverse-light dark cycle) and habituated for 7–15 days prior to surgery with ad libitum access to food and water. Intravenous surgery was performed as per our previous publication [10,11,15]. Briefly, rats were anesthetized with ketamine and xylazine (50 and 5 mg/kg, i.p., respectively); one end of a silastic catheter was placed in their right jugular veins while the other end of the catheter was attached to a modified 22-gauge cannula, which was mounted to the backs of the rats. After the surgery, rats were injected with meloxicam (1 mg/kg, s.c.) to relieve pain and were allowed to recuperate for 5 to 7 days before the start of self-administration (SA). Experimental procedures were conducted in accordance with the guidelines of the *Guide for the Care and Use of Laboratory Animals: Eighth Edition* (ISBN 0-309-05377-3). The NIDA Animal Care and Use Committee approved the study protocol (23-MNPB-9).

2. METH Self-Administration and Footshock

Drug-naive rats were allowed to SA METH (0.1 mg/kg/infusion) and saline in three 3 h sessions (9 h/day) with a 30 min time interval between each session under an FR1 schedule, with a 20-s timeout between each infusion. Throughout the experimental period, rats were singly housed with free access to food and water. Rats were trained for 5 weekdays with 2 weekend days off. During off days, rats were housed in the SA chambers disconnected from the intravenous SA lines. After 20 days of SA, METH self-administered rats were subjected to footshock (aversive stimuli) for the next 11 days, during which 50% of the reinforced active lever presses resulted in a contingent delivery of a footshock (0.5 s) through the grid floor. The footshock intensity was progressively increased from 0.18 mA to 0.42 mA. At the end of the footshock phase, some of the rats continued to take METH despite aversive stimuli and were termed as “shock resistant (SR) rats”, while rats that suppressed the METH intake were termed as “shock sensitive (SS)”. The SR and SS rats were separated as per our previous published studies [10–15]; specifically, animals were classified as shock-sensitive if they reduced their intake by 60%. Control rats did not receive foot-hocks.

3. Incubation of Drug Craving

After post-training, punishment, SCH23390 treatment, and the resurgence phase, rats were housed in their home cage in the animal vivarium for a period of 30 days. On withdrawal days (WD) 2 and 30, a cue-induced METH-seeking test was carried out. Rats were brought back to their respective SA chambers, and pressing on the "active" lever resulted in contingent demonstration of the tone and light cues but no METH infusions. A cue-induced drug-seeking behavior test was carried out for one 3 h session. Animals were euthanized 24 h after WD30, and the dorsal striatal tissue samples were extracted.

4. RNA Extraction and Sequencing

Messenger RNA was extracted from 1 ug of total RNA using the NEBNext Poly(A) mRNA Magnetic Isolation Module (New England Biolabs, Ipswich, MA, USA). RNA sequencing libraries were generated using the NEBNext Ultra II RNA Library Prep kit following the manufacturer's instructions (New England Biolabs, Ipswich, MA, USA). Briefly, enriched mRNAs were fragmented for 15 min at 94 °C. First strand and second strand cDNAs were subsequently synthesized. cDNA fragments were end repaired and adenylated at 3′ ends, and universal adapters were ligated to cDNA fragments, followed by index addition and library enrichment by limited-cycle PCR. The sequencing libraries were then validated on the Agilent TapeStation 4200 (Agilent Technologies, Palo Alto, CA, USA) and quantified by using the Qubit 2.0 Fluorometer (Invitrogen, Carlsbad, CA) as well as by quantitative PCR (KAPA Biosystems, Wilmington, MA, USA). The sequencing libraries were clustered on two flow cell lanes. After clustering, the flow cells were loaded on the Illumina® HiSeq instrument (4000 or equivalent) according to the manufacturer's instructions. The samples were sequenced using a 2 × 150 bp paired-end (PE) configuration (GeneWiz, South Plainfield, NJ, USA). Image analysis and base calling were conducted by the HiSeq Control Software (HCS). Raw sequence data (.bcl files) generated from Illumina HiSeq was converted into fastq files and de-multiplexed using Illumina's bcl2fastq 2.17 software. One mismatch was allowed for index sequence identification.

After investigating the quality of the raw data, sequence reads were trimmed to remove possible adapter sequences and nucleotides with poor quality using Trimmomatic v.0.36. The trimmed reads were mapped to the Rattus norvegicus reference genome available on ENSEMBL using the STAR aligner v.2.5.2b. The STAR aligner is a splice aligner that detects splice junctions and incorporates them to help align the entire read sequence. BAM files were generated as a result of this step. Unique gene hit counts were calculated by using feature counts from the Subread package v.1.5.2. Only unique reads that fell within exon regions were counted. After extraction of gene hit counts, the gene hit counts table was used for downstream differential expression analysis. Using DESeq2, a comparison of gene expression between the groups of samples was performed. The Wald test was used to generate p-values and log2 fold changes. Genes with adjusted p-values < 0.05 and absolute log2 fold changes > 1 were called differentially expressed genes (DEGs) for each comparison. A gene ontology analysis was performed on the statistically significant set of genes by implementing the software gene set clustering based on functional annotation (GeneSCF).

5. Western Blot

Dorsal striatal tissues from the other hemisphere were homogenized using 10 mM Tris HCl, 150 mM NaCl, pH 7.5 in the presence of 1% Nonidet P-40 (NP-40) protein and phosphatase inhibitor cocktails (Sigma, St. Louis, MO). Total protein concentrations were quantified using the BCA assay (Thermofisher Scientific, Waltham, MA). Twenty μg of soluble cytosolic protein lysate were prepared in solutions that contained Laemmli buffer and 5% β-mercaptoethanol. Samples were then boiled at 70 °C for 10 min, put in ice, and

resolved using NuPage 10% Bis-Tris Protein Gels (ThermoFisher Scientific, Waltham, MA). Proteins were electrophoretically transferred onto PVDF membranes (Bio-Rad, Hercules, CA). Membranes were blocked with 5% Blotting-Grade Blocker (Bio-Rad, Hercules, CA) in TBST for two hours, briefly washed, then were re-blocked with 5% BSA Sigma (Saint Louis, MO, USA) in TBST and incubated overnight with primary antibodies at dilutions described by the manufacturer. Primary rabbit polyclonal antibodies, including anti-CPEB4 (# 28748S); CAMKII-α (1:1000, #3357); and eIF4e (1:1000, #9742) were purchased from Cell Signaling Technologies (Danvers, MA, USA). Anti-CPEB1 (1:1000, # PA1-1100), CPEB2 (1:1000, # PA1-113055), GLD2 (1:1000, # PA5-69570); AUORA-A (1:1000, # PA5-86309); TACC3 (1:1000, # PA5-36349); and PABP (1:1000, # PA5-29883) were purchased from Invitrogen (Waltham, MA, USA). Anti-CDK1 (1:1000, #19532-1-AP); CPSF2 (1:1000, #17739-1-AP); PARN (1:1000, #13799-1-AP); and Symplekin (1:1000, #11519-1-AP) were purchased from ProteinTech Group, Inc (Rosemont, IL, USA). Cyclophilin rabbit polyclonal antibody (1:10,000, AB16045) and CPEB3 (1:1000, AB10833) were purchased from Abcam (Waltham, MA, USA). Anti-α-tubulin (1:10,000; # T6074) and NR2A (1:1000, # 07-632) were purchased from Millipore-Sigma (Burlington, MA, USA). The antibodies revealed bands at the expected molecular weights for all proteins. Anti-rabbit HRP (1:6000 #7074) and anti-mouse HRP (1:6000 #7076) secondary antibodies were purchased from Cell Signaling Technologies (Danvers, MA, USA). After secondary antibody incubation, ECL Clarity (Bio-Rad, Hercules, CA) was used to detect bands on the ChemiDoc Touch Imaging System (Bio-Rad, Hercules, CA), and intensities were measured with Image Lab 6.0 version (Bio-Rad, Hercules, CA) software.

6. Statistical Analysis

Behavioral data were analyzed with the statistical program GraphPad Prism (version9, GraphPad software, La Jolla, CA, USA). To understand the nature of interactions further, 2-way ANOVA with repeated-measures analyses was also undertaken to compare the groups. Variables were numbers of METH infusions on training days, between-subject factors (CT, SR, and SS), and within-subject factor SA days (training days 1–20), and their interactions. Bonferroni post hoc tests were used to reveal the significant differences. METH-seeking data were also analyzed using 2-way ANOVA with repeated measures followed by Bonferroni post hoc tests, with variables being group (CT vs. SR or SS) and withdrawal days (WD2 and WD30). The quantitative RT-PCR and Western blot data were analyzed by one-way ANOVA followed by Tukey's post hoc test using GraphPad Prism (version9). The null hypothesis was rejected at $p < 0.05$.

Table S1. List of RT qPCR primer sequences.

Gene Name	Forward Primer	Reverse Primer
B2m	GAT CTT TCT GGT GCT TGT	AGC TCA ATT TCT ATT TGA GGT
Chm	CTC TAC TTC AAT ATG AGA GAC TCT T	TGC ATT ATC ATT TCC TAG ACC G
Cpeb1	GCC ATC TTG AAT GAC CTA TTT	GTT GGT TAT TGA AAG TCA CAC G
Cpeb2	ATG TTA TAG CAC CAC CGA	GTG TTA CTA TTG TTG TCT GTC C
Cpeb3	GAC CCC TTC GAG CTG TTG AA	GCT GAT GGC TGC AAT GTA GC
Cpeb4	CCC GGA ATT GAA GTA GCA TCA ATC	AGC AGG TAA TGA GCT ACT TTC CAA
Foxn2	ACA TAG AGG GAG ATG ATA TGC	TGG CCA TGT AAA TGA GAA GAC
Glun2a/NR2a	CTA TGT GGA GAG AGG CTG CG	AAG GTG GAG GAT GCA ATG GG
Creb1	TAC AAA CAT ACC AGA TTC GCA	CAT ATT CTT TCT TCT TTC TAC GAC
Cbp/Kat3a	TGG ACT GAA TAC GGC AC	ACT ATT GAC CAT GCT CTG
JunD	CCT GGA GGA GAA AGT CAA GAC C	GTG GCT GAG GAC TTT CTG TTT G

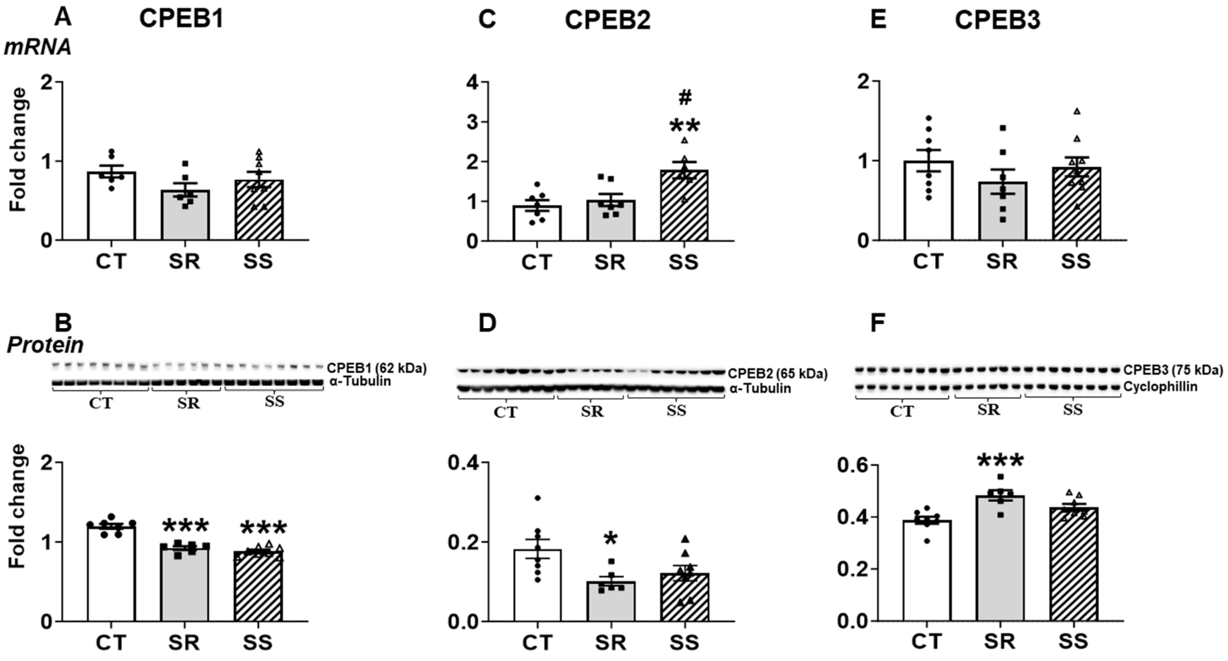

Figure S1. mRNA and protein expression analysis of CPEB family members. (**A**) and (**B**) CPEB1; (**C**) and (**D**) CPEB2; (**E**) and (**F**) CPEB3, respectively. Keys to statistics CT, saline; SR, shock-resistant; and SS, sensitive rats. Key to statistics: **p* < 0.05, ***p* < 0.01, ****p* < 0.001, comparisons between METH groups (SR and SS) and controls (CT); #*p* < 0.05, ##*p* < 0.01, ###*p* < 0.001, comparison between SR and SS.

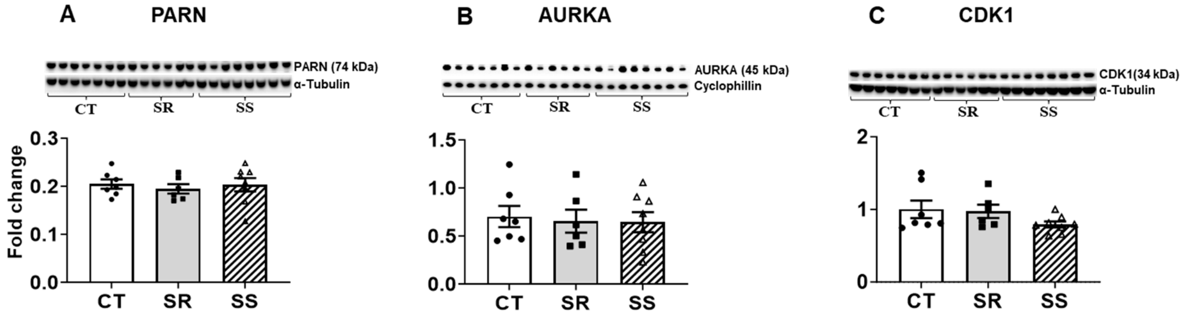

Figure S2. Protein expression analysis of (**A**) PARN, (**B**) AURKA, and (**C**) CDK1.
